# A Rare Case of Cholesterol Granuloma as a Complication of Onyx Embolization for Dural Arteriovenous Fistula

**DOI:** 10.1007/s00270-025-03972-5

**Published:** 2025-02-06

**Authors:** Ibrahim Ghanem, Nicole Rotter, Claudia Scherl, Johannes Ludwig, Bruno Reible, Angela Schell

**Affiliations:** 1 Department of Otolaryngology, Head and Neck Surgery, University Hospital Mannheim, Theodor-Kutzer-Ufer 1-3, 68167 Mannheim, Germany; 2 Department of Radiology, University Hospital Mannheim, Mannheim, Germany; 3 Department of Pathology, University Hospital Mannheim, Mannheim, Germany

To the Editor:

Cholesterol granuloma (CG) is both a clinical and pathological diagnosis, signifying a tissue response within the temporal bone due to the presence of a specific foreign element cholesterol crystals [1].

CGs are commonly associated with conditions such as serous otitis media, chronic otitis media, prior otologic surgery, or localized trauma [2].

A 69-year-old patient presented to our clinic in 2021 with a history of catheter embolization using the Onyx Liquid Embolic System in 2009 to address a right temporal arteriovenous dural fistula (DAVF). Over the past year, the patient reported right-sided retroauricular pain, headaches, and bilateral hearing loss, more pronounced on the right. The otoscopic examination of the right ear revealed just a retraction pocket in the epitympanum. Pure-tone audiometry showed: Left ear with moderately severe sensorineural hearing loss; right ear with moderately severe mixed hearing loss and a conductive component, maximum loss of 20 dB.

**Fig. 1:**
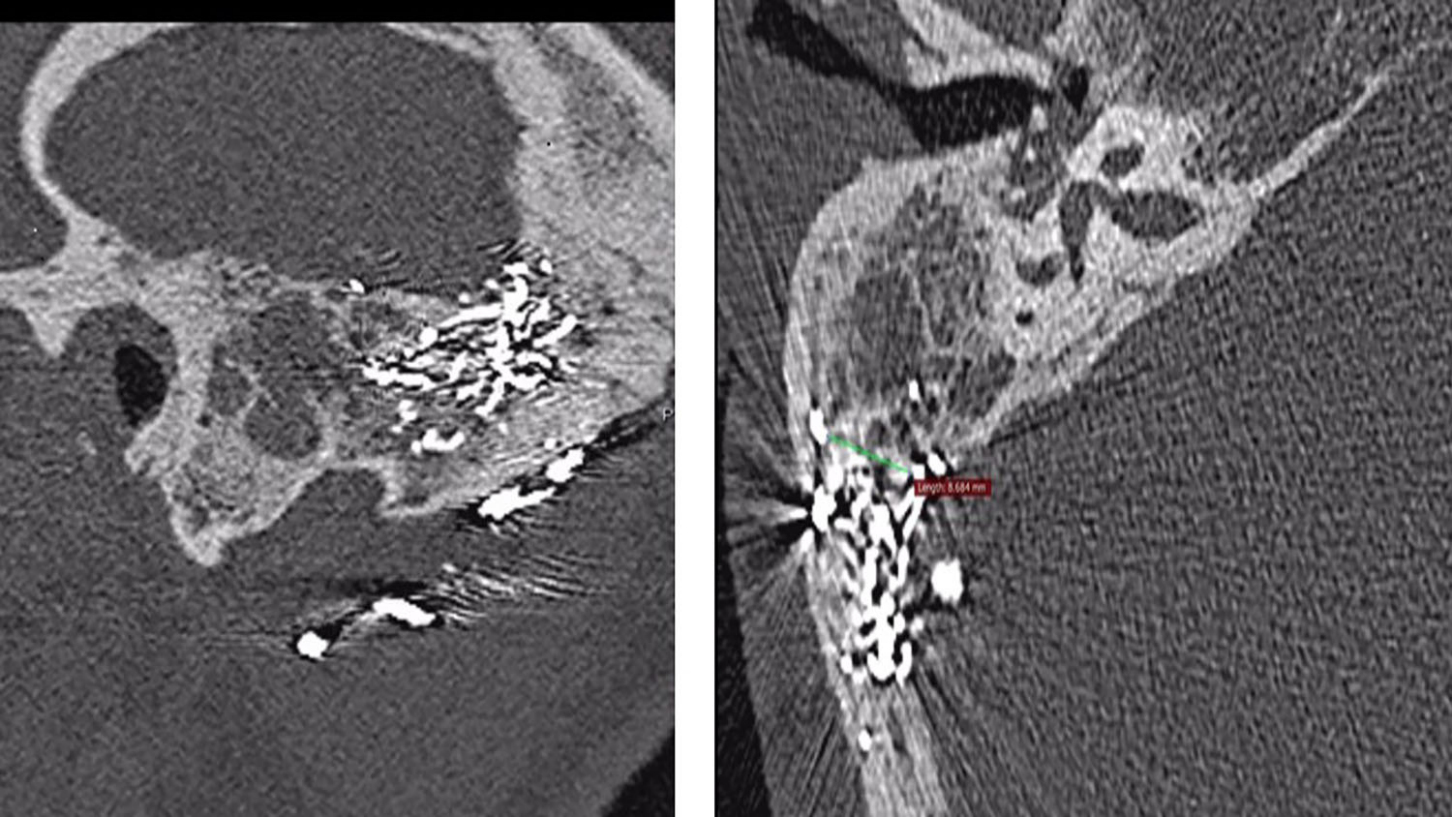
High-resolution CT axial and sagittal imaging of the temporal bones: Onyx in the temporal bone and in the extracranial venous circulation

The angiography-MRI scan revealed signs of mastoiditis and opacity of the mastoid cells in the right ear, but no evidence of recurrent dural fistula. High-resolution CT axial imaging of the temporal bones (with 0.75 mm slices) raised suspicion of a cholesteatoma with subtotal opacification of the mastoid and several metallic, rounded structures in the dorsal mastoid, extending into the temporal skull vault and the right nuchal soft tissues. The estimated migration distance between the temporal DAVF and the affected mastoid cells is a maximum of approx. 8.7 mm (Fig. 1). The left mastoid cells and middle ear showed no pathology. Angiography of the cerebral vessels showed no evidence of a DAVF.

**Fig. 2:**
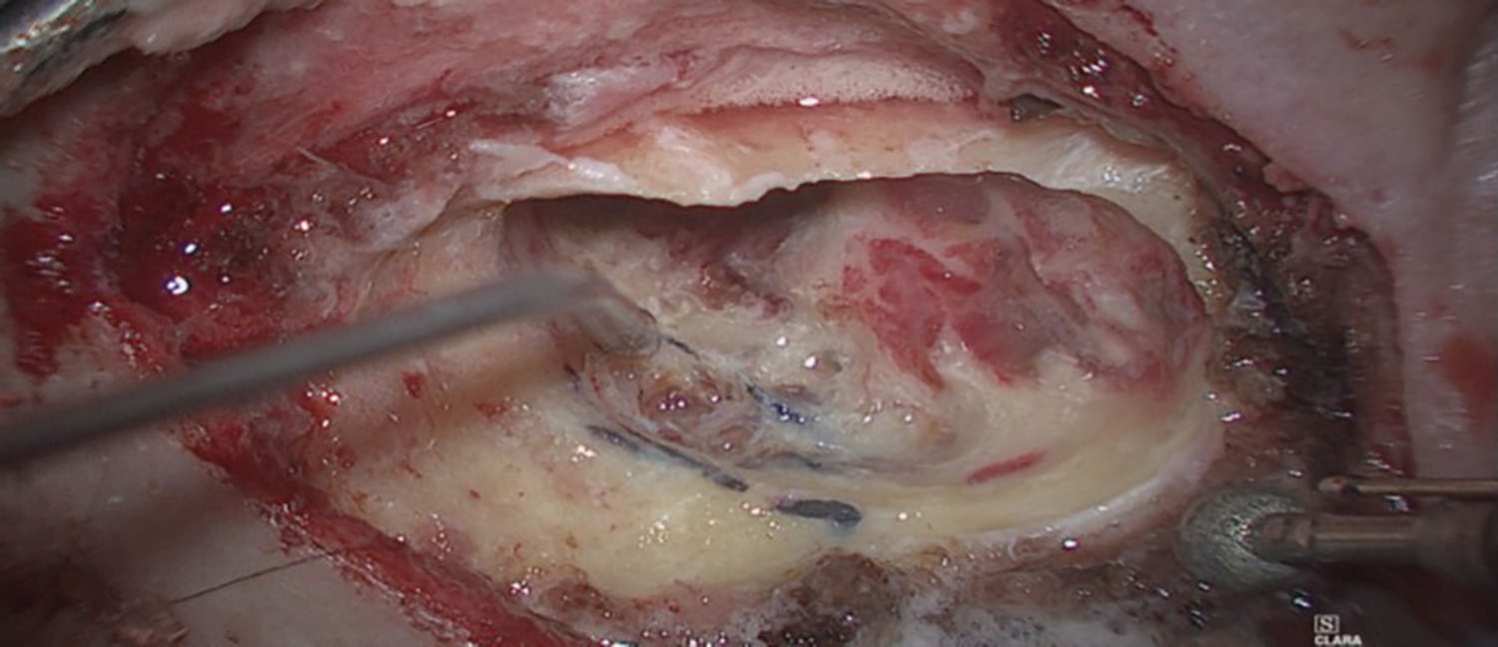
Intraoperative image of the right mastoid

The surgery was performed via a postauricular approach. Early in the mastoidectomy, scattered foreign material resembling the Onyx substance, along with hyperplastic mucosa and generalized inflammatory changes in the bony structures (Fig. 2), was promptly identified and exposed using a diamond drill. Further dissection along the dura and toward the sigmoid sinus revealed additional similar foreign bodies, which were carefully removed.

The patient was discharged on the third postoperative day and recovered quickly without pain, headaches, or facial weakness. Follow-up over nearly two years shows the ear and mastoid area remain healthy and free from granulation or abnormalities.

**Fig. 3:**
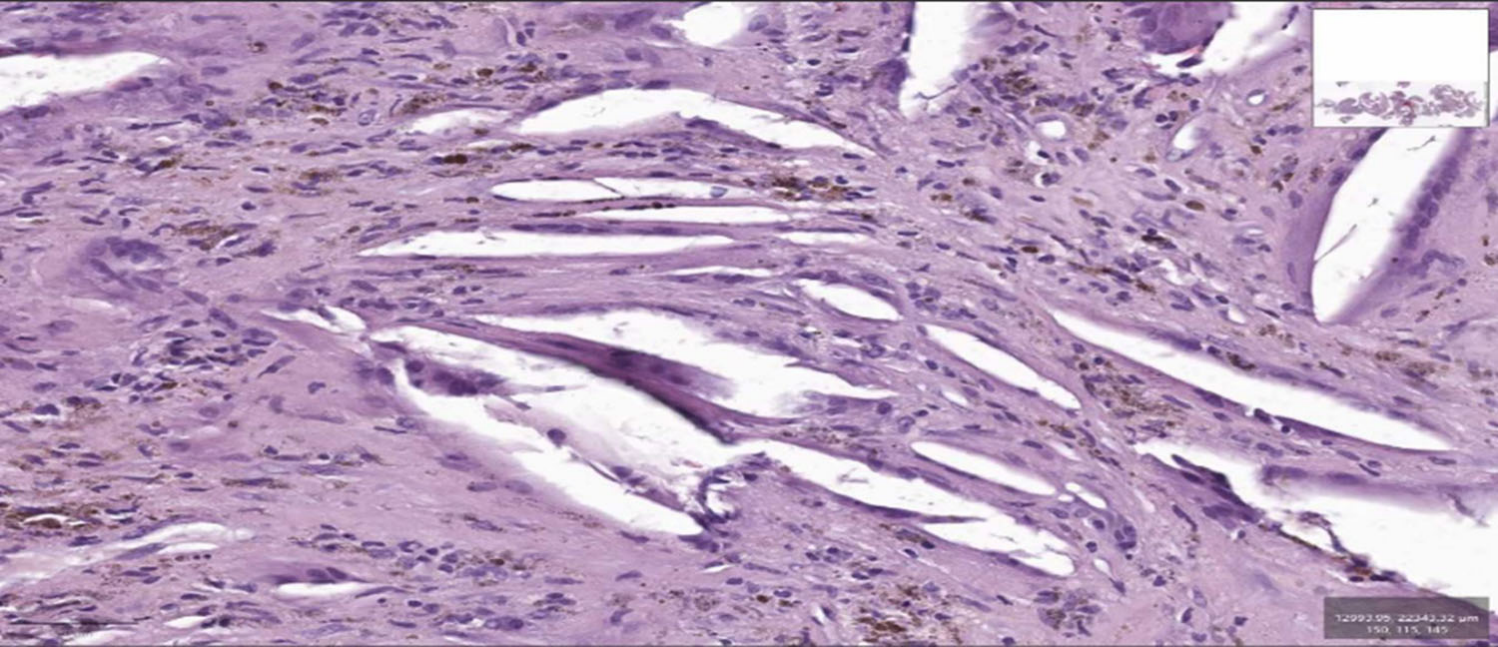
display tissue samples removed from the mastoid during surgery, thus confirming the diagnosis of cholesterol granuloma resulting from the migration of embolization material into the mastoid bone

Histopathologic examination revealed fibrous tissue surrounded by abundant cholesterol crystals, multinucleated giant cells of foreign body type, and areas densely populated with hemosiderin-laden macrophages (Fig. 3). DAVFs represent 10–15% of all intracranial vascular malformations, primarily occurring near the sigmoid/transverse sinus. Treatment options include neurosurgical resection and endovascular embolization with Onyx, which has become the treatment of choice due to its high success and safety rate and low complications [3].

The most common complications associated with this procedure typically include intracranial hemorrhage (ICH), ischemia, venous infarction, and cranial nerve palsy. Complications such as CG formation and chronic mastoiditis due to the migration of Onyx materials after embolization have not been widely documented [4].

The management of CG in this region typically leans towards a conservative approach, and surgery is not always necessary [5]. In our case, due to the severity of the patient’s symptoms and lack of improvement with conservative treatment, surgery was performed. This approach led to successful treatment without complications, and over the following two years, there was no recurrence of primary symptoms (tinnitus) or secondary symptoms (retroauricular pain/headaches).

This case represents a rare occurrence of a cholesterol granuloma following Onyx embolization of a DAVF. The successful surgical management highlights the need for further investigation into the management of foreign material migration into the mastoid region.
